# Interplay of *PNPLA3* and *HSD17B13* Variants in Modulating the Risk of Hepatocellular Carcinoma among Hepatitis C Patients

**DOI:** 10.1155/2020/4216451

**Published:** 2020-04-24

**Authors:** Carla De Benedittis, Mattia Bellan, Martina Crevola, Elena Boin, Matteo Nazzareno Barbaglia, Venkata Ramana Mallela, Paolo Ravanini, Elisa Ceriani, Stefano Fangazio, Pier Paolo Sainaghi, Michela Emma Burlone, Rosalba Minisini, Mario Pirisi

**Affiliations:** ^1^Department of Translational Medicine, Università del Piemonte Orientale (UPO), Novara, Italy; ^2^Department of Medicine, AOU Maggiore della Carità, Novara, Italy; ^3^AOU Maggiore della Carità, Virology Unit, Novara, Italy

## Abstract

A single-nucleotide polymorphism causing a C to G change in the *PNPLA3* gene (rs738409) is associated with disease severity and development of hepatocellular carcinoma (HCC) in nonalcoholic fatty liver disease; the insertion variant rs72613567:TA of the 17*β*-hydroxysteroid dehydrogenase type 13 (*HSD17B13*) mitigates this detrimental effect. Our aim was to evaluate if the same holds true in chronic hepatitis C virus infection (HCV). With a case control retrospective study design, we selected 110 patients who developed HCC on a background of HCV infection, matching each patient for sex and age (±30 months) to three HCV-infected, non-HCC patients. All participants underwent genotyping for *PNPLA3* and *HSD17B13* gene variants. Both univariate and multivariate analyses of risk factors for advanced disease and HCC were performed. Carriage of *PNPLA3* G∗ allele was associated with a trend of progressively more severe liver disease, from mild fibrosis to significant fibrosis, cirrhosis, and HCC (*p* = 0.007). When the *HSD17B13:TA* status of these patients was taken into account, the abovementioned trend was strengthened among *HSD17B13* major allele homozygotes and completely blunted among carriers of the minor allele (*p* = 0.0003 and 0.953, respectively). In a conditional logistic regression model including diabetes and AST to platelet ratio index among predictor variables, the unfavourable genetic profile characterized by the coexistence of the *PNPLA3* minor allele and *HSD17B13* major allele (vs. all other possible combinations) was an independent risk factor for HCC (OR = 2.00, 95% CI: 1.23-3.26) together with a history of alcohol abuse. In conclusion, carriage of the combination *PNPLA3* minor allele and *HSD17B13* major allele may represent a risk factor for HCC among HCV-infected patients. The interplay between the two genes may explain some of the controversy on this topic and may be exploited to stratify HCC risk in hepatitis C.

## 1. Introduction

The natural history of chronic liver diseases is highly variable, ranging from minimal histological damage to cirrhosis and hepatocellular carcinoma (HCC) [1, 2]. Some of this variability is genetically determined. The carriage of the G allele in the single-nucleotide polymorphism (SNP) I148M (rs738409) of the adiponutrin gene (*PNPLA3*) on a background of metabolic, alcoholic, and viral disease is one of the best examples of a gene variant affecting the natural history of chronic liver diseases [3]. In fact, carriers of the G allele at the abovementioned locus have a reduction in the enzymatic activity of adiponutrin, resulting in higher intracellular triglyceride levels [3], which might then increase their risk to develop advanced fibrosis, cirrhosis, and HCC. As far as hepatitis C virus- (HCV-) infected patients are concerned, however, the association between *PNPLA3* variants and HCC has been debated, suggesting that, in this condition, other factors may mask the alleged effect on hepatocarcinogenesis [4, 5].

Recently, a protein-truncating variant responsible for a loss-of-function mutation of in the *HSD17B13* gene (rs72613567) has been associated with a decrease in aminotransferase levels and a reduction of liver injury in the setting of a fatty liver, mitigating the risk conferred by the carriage of *PNPLA3* I148M variant [6, 7]. *HSD17B13* codes for a retinol dehydrogenase, 17*β*-hydroxysteroid dehydrogenase type 13, which is highly expressed in the liver. Although the physiological role of this protein has not been fully elucidated yet, it appears to be involved in lipid biosynthesis, as well as in the modulation of sex hormone, bile acid, and fatty acid redox processes [8, 9]. Importantly, the truncated variant has been shown to be protective against HCC development among patients with alcoholic liver disease [10].

Since alcoholic and nonalcoholic fatty livers have an impact on the natural history of HCV infection [11], we wondered whether carriage of *HSD17B13:TA*, by modulating the detrimental effects of carrying an unfavorable *PNPLA3* genotype, may protect against cirrhosis and HCC development in patients with long-standing HCV infection.

## 2. Methods

### 2.1. Study Population

We designed a case-control study, including *N* = 440 patients attending the liver clinic of an academic hospital in northern Italy, affected by long-standing chronic HCV infection at different stages. The study has been conducted in strict accordance with the principles of the Declaration of Helsinki; all the patients gave an informed consent to their participation to the study, which was approved by the local ethical committee (Comitato Etico Interaziendale di Novara, no. 176/18).

Because timing of infection is hard to determine, we chose to include only patients aged 60 years or older, thus reducing the probability of a duration of infection shorter than 20 years. Therefore, we applied the following inclusion criteria: age ≥ 60 years and laboratory evidence of chronic HCV infection.

The only exclusion criterion was the inability to give informed consent to participation in the study.

To assess the role of rs72613567:TA in liver disease progression, we considered *N* = 110 patients with HCV-related HCC of any-BCLC (Barcelona Clinic Liver Cancer) stage [12]. For each of them, we selected three sex- and age- (±30 months) matched controls among patients affected by chronic HCV infection without HCC development, referred to the liver clinic for the diagnosis and treatment of their condition.

We collected demographic, clinical, and biochemical data simultaneously to blood sampling.

### 2.2. HCC Diagnosis

The diagnosis of HCC has been made by imaging (computed-tomography scan or magnetic resonance imaging) [13] and was confirmed by a liver biopsy when clinically appropriate, according to EASL Clinical Practice Guidelines [12]. All the HCC patients included liver cancer superimposed to cirrhosis.

### 2.3. Staging of Controls

Non-HCC patients were classified according to the METAVIR staging system through transient elastography (FibroScan®) [14]. To define the absence of significant fibrosis, we used the cut-off value of 7.1 kPa (F1); a threshold of 12.5 kPa was chosen to define cirrhosis (F4). Patients with a liver stiffness (LS) between 7.1 and 12.4 were lumped in the F2-F3 group [15]. Whenever a valid LS value was not available (*N* = 9/330, 2.7%), we used the AST to platelet ratio index (APRI) to stage liver fibrosis (APRI < 0.7 = F1; APRI 0.1‐1 = F2/F3; APRI > 1 cirrhosis) [16]. By doing so, *N* = 5/9 patients were considered cirrhotic and *N* = 3/9 were classified as F1 and *N* = 1/9 as F2-F3.

### 2.4. Genetic Studies

Genomic DNA was extracted from whole blood or buffy coat, using a commercial kit (Invitrogen, Carlsbad, CA, USA), according to the manufacturer's instructions. DNA was then amplified by polymerase chain reaction (PCR). The PCR primer sequences used for *PNPLA3* amplification were as follows: forward: 5′-CCTGCAGGCAGGAGATGTGT-3′; reverse: 5′-GCCCTGCTCACTTGGAGAAA-3′. The PCR primer sequences used for *HSD17B13* amplification were as follows: forward: 5′-GTCTGAGGCATGAGAATTGCT-3′; reverse: 5′-GGCCTGTATTGGAGACAGATG-3′. To define the genotype of the two target genes, we performed a restriction fragment length PCR. NLA-III and TRU1I restriction enzymes (Life Technologies, Thermo Fisher Scientific; Carlsbad, California, US) were used to digest *PNPLA3* and *HSD17B13*, respectively. All samples were amplified twice; when discordant, they were run the third time. The lab technicians performing the genetic studies were blinded about the case/control state of the subjects enrolled.

### 2.5. Statistical Analysis

Statistical analysis of data was carried out with the software package Stata, version 13.1 (StataCorp LP, College Station, Texas, US). The measures of centrality and dispersion of data chosen were medians and interquartile ranges. Continuous variables were compared between groups by the Mann–Whitney test; the nonparametric test chosen to identify a trend across ordered groups was that developed by Cuzick. Exact Fischer's test and Pearson's chi-square test were used, as appropriate, to explore the associations of categorical variables. Moreover, to better understand the relationship between severity of liver disease (in four ordered categories: mild, moderate, or advanced fibrosis and HCC) and variants of the two genes of interest, we run two ordered logistic models (one with and one without an interaction term), followed by a likelihood ratio test to evaluate whether these two models were statistically different.

The *χ*^2^*G* test “Goodness of Fit” was employed to verify whether the proportions of the two polymorphisms were distributed in patients in accordance with the Hardy-Weinberg equation. Finally, a conditional logistic regression analysis was performed to test the independency of the association between the genes of interest and HCC development.

The level of significance chosen for all statistical tests was 0.05 (two-tailed).

## 3. Results

### 3.1. Characteristics of the Study Population

Table 1 presents the main demographic, anthropometric, and clinical characteristics of the study population. Cases and controls were well-matched with regard to age and sex and were of similar body mass index. Diabetics and individuals with a history of excess alcohol consumption (as defined by the Italian drinking guidelines for the general population) [17] were overrepresented among cases. Also, AST levels were slightly, but significantly, higher among cases. Lastly, HCC patients showed a significantly higher liver fibrosis with either of the two proxy measures (LS, APRI) we used. The characteristics of controls when categorized in three subgroups based on METAVIR stage are shown in Supplementary Table 1.

### 3.2. Genotype and Allele Frequencies for rs738409 and rs72613567

**Figure 1:**
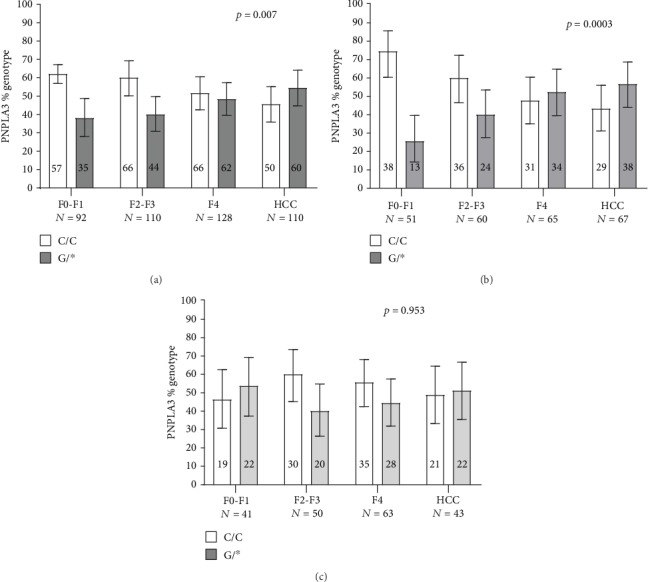
Proportions of rs738409 major allele homozygotes (white bars) and G/∗ allele carriers (colour bars), among controls with progressively more advanced METAVIR stages and in HCC cases. (a) Entire population; (b) major allele homozygotes for *HSD17B13:TA*; (c) carriers of the HSD17B13:TA variant allele. Error bars represent 95% confidence intervals.

Table 2 shows genotype and allele frequencies of the rs738409 (*PNPLA3*) SNP and the insertion variant rs72613567 (*HSD17B13*) in the study population. The proportion of patients who carried the rs738409 G allele increased moving from mild to moderate fibrosis, to cirrhosis, and to HCC. This trend was far stronger among rs72613567 major allele homozygotes and completely blunted among carriers of the rs72613567 insertion variant allele (Figure 1). Moreover, in the first of two ordered logistic models having severity of liver disease as an outcome variable and variants of the genes of interest (without an interaction term) as predictor variables, the likelihood ratio chi-square test was 7.62 (*p* = 0.022). In the second model, also having severity of liver disease as an outcome variable but including as predictor variables variants of the genes of interest plus an interaction term between them, the likelihood ratio chi-square test was 13.9 (*p* = 0.011). When compared with a likelihood ratio test, the two models were statistically different (*p* = 0.012), confirming the importance of the interaction between the two genes.

Carriers of the rs738409 G allele who also carried the major allele for rs72613567, the combination shown to increase the risk of developing fibrosis in the presence of a fatty liver, were *N* = 109/440 (25%). The proportion of patients with such “high-risk” combination was significantly higher than that of patients with HCC compared to age- and sex-matched controls without HCC (38/110, 35% vs. 71/330, 22%, *p* = 0.007).

### 3.3. Logistic Regression Analysis Models for HCC

At conditional logistic regression analysis, being a carrier of the dummy variable “high-risk genetic combination” (1 = carriage of both rs738409 minor allele and rs72613567 major allele; 0 = all other combinations between the two genes of interest) and having a history of alcohol abuse were predictors of HCC independent of diabetes and AST to platelet ratio index.  Table 3 summarizes the results of such logistic model (*N* of observations = 440). Running two further conditional logistic regression models (i.e., substituting the dummy variable “high-risk genetic combination” either with each variant of the genes of interest, or with an interaction term) demonstrated only a nonsignificant trend (*p* = 0.061) for the stratum of patients corresponding to carriers of the “high-risk combination”, but the likelihood ratio test did not detect any significant difference between the two models (*p* = 0.355).

## 4. Discussion

The main finding of the present study is the demonstration that specific variants of *HSD17B13* and *PNPLA3* genes interplay in the determination of genetic predisposition to chronic liver disease progression and liver cancer in HCV-infected subjects. These data will be discussed at the light of the current literature on this topic.

Chronic HCV infection remains a leading cause of chronic liver diseases (CLD) worldwide [18], despite the high rates of sustained viral response obtained with direct antiviral agents [19]. The burden of long-standing HCV-infected patients who have developed significant liver damage constitutes an important target for surveillance, also after having achieved viral clearance [20]. Indeed, in cross-sectional and case-control studies, HCV infection is associated with a 15- to 20-fold risk of developing HCC compared with HCV-negative subjects [21].

Liver carcinogenesis is the result of a multistep process, in which the main risk factor is the presence of chronic liver damage leading to persistent inflammation [2]. However, in the last years, genome-wide association studies have identified some genetic variants potentially involved in liver disease progression and, eventually, in HCC development [3, 10, 22]. Among them, the best validated relationship is that occurring between the I148M variant of adiponutrin-coding gene and CLD of different etiologies [3]. Specifically looking to HCV infection, *PNPLA3* I148M variant has been related to an increased risk of liver fibrosis progression [23]. Although the role of *PNPLA3* mutation in fibrogenesis is widely acknowledged, the underlying mechanisms are poorly understood. What is known is that rs738409 C>G variant causes the accumulation of the mutated protein on the surface of lipid droplets, impairing the mobilization of triglycerides [24, 25]. Furthermore, in humans, this missense mutation also leads to a deranged fat efflux from the liver [26] and to impaired hydrolysis of retinyl esters in hepatic stellate cells [27]. As a result, lipid turnover is deeply altered, and this may favor persistence of inflammatory trigger(s).

The evidence on an association between *PNPLA3* mutation and HCC is less striking. In fact, while the rs738409 mutation has been convincingly associated to liver carcinogenesis in alcoholic liver disease [28, 29] and in nonalcoholic steatohepatitis [30, 31], this association in viral hepatitis has been the subject of controversy [32, 33]. While some authors reported an increased risk of HCC development in HCV patients carrying the G allele [34–38], others failed to confirm these findings [4, 39–42]. The reason for this discrepancy may belong to the different ethnicities of the study populations; in fact, the largest part of the studies failing to demonstrate the association of this SNP with HCC has been conducted on Asian populations. In a very recent meta-analysis, the association between HCC and rs738409 has been confirmed in Caucasians [5]. This is in line with other reports, according to which carriage of the *PNPLA3* mutation is a risk factor for liver disease progression and HCC in Caucasians but not in Asians [23, 43]. Taking these findings together, it is reasonable to postulate that carriage of the G allele in the rs738409 polymorphism contributes to both fibrogenesis and liver carcinogenesis, but other genetic contributors might modulate its effect, thus explaining the differences observed among ethnic groups.

In 2017, Abul-Husn et al. firstly reported that the loss of function variant *HSD17B13:TA* mitigates the risk of liver disease progression conferred by carriage of *PNPLA3* I148M among NAFLD patients [6]. Further recent data suggest a potential protective role of *HSD17B13:TA* with regard to CLD progression in HCV infection [44], although its interplay with *PNPLA3* polymorphism has not been investigated in this context. Our data are consistent with both studies. In fact, in a population of chronic HCV-infected patients, the prevalence of rs738409 C>G variant of adiponutrin gene increases for progressive degrees of liver fibrosis, being lowest for mild fibrosis and highest in patients affected by cirrhosis. Even more strikingly, this trend is abolished by the concomitant presence of *HSD17B13:TA* allele and strengthened in *HSD17B13* major allele homozygotes, with a significant effect of the interaction between the two genes. This is in line with the hypothesis that the loss of function of *HSD17B13* might be protective against the increased risk of liver fibrosis progression attributable to adiponutrin mutation. The mechanisms of the interplay between these two proteins are still unknown, though they are probably related to lipid turnover. In fact, *HSD17B13* encodes a protein localized on the surface of lipid droplets, which is upregulated in the livers of patients and mice with NAFLD. Its overexpression is associated to an increase in the number and size of lipid droplets. Consistent with this interpretation, the hepatic overexpression of *HSD17B13* in murine models significantly increases lipogenesis and triglyceride content in the liver, leading to a fatty liver phenotype [45]. The *HSD17B13:TA* variant results in a truncated protein with reduced enzymatic activity [46].

The second question that we tried to address was whether carrying the allele *HSD17B13:TA* is also protective against HCC development in HCV patients. In a recent study by Yang et al., the presence of *HSD17B13:TA* was protective against HCC in alcoholic liver disease, but not in HCV-infected subjects; however, the authors did not analyze the contribution of *PNPLA3* to the overall genetic risk [10]. According to our data, the protective effect of *HSD17B13:TA* is extended to HCC. In fact, when HCC and non-HCC patients are compared, the strict association between the G allele of *PNPLA3* and the diagnosis of HCC is lost in the group of patients carrying at least one *HSD17B13* minor allele.

In a conditional logistic regression model, we confirmed the independent contribution given by the combination of the two gene variants to the risk of developing HCC along the course of HCV infection. The coexistence of *PNPLA3* G allele and *HSD17B13* major allele homozygosity is an unfavourable, high-risk genetic profile for HCC, which is independent from alcohol abuse, degree of fibrosis, and diagnosis of diabetes mellitus. Thus, the association with hepatocarcinogenesis may not to be entirely accounted for by the consequences of accelerated fibrogenesis that—not unexpectedly—do play a major role. A clinical implication of these findings relates to the potential application of genotyping to screening strategies. Risk stratification for HCC development may take advantage, in the future, from gene-based prediction models, which might find a place in clinical practice.

A further interesting finding emerging from our conditional regression analysis is the independent contribution of alcohol abuse to liver cancer in HCV-infected subjects. There is ample evidence that alcohol synergistically contributes to the detrimental viral insult on liver disease progression [47, 48]. HCV-infected patients are already discouraged from alcohol consumption: whether this needs to be reinforced in the presence of an unfavourable genetic profile, even after resolution of HCV infection, remains to be ascertained.

The retrospective, cross-sectional design of the present paper does not allow us to infer any causal relationship. Moreover, the fact that the association between the two genes of interest and HCC did not reach statistical significance when all their combinations were tested together suggests that our findings await confirmation in larger studies on prospectively recruited patients. However, to our knowledge, this is the first study designed to test the interplay between *PNPLA3* and *HSD17B13* on a homogeneous cohort of long-standing HCV-infected patients, being controlled for two major factors influencing the course of HCV infection, i.e., age and sex.

In conclusion, *HSD17B13:TA* mitigates the increased risk of liver fibrosis progression and HCC in HCV-infected patients with *PNPLA3* I148M variant; further studies are required to better elucidate the underlying pathogenic mechanisms and to evaluate the potential clinical applications of this observation.

## Figures and Tables

**Table 1:** Main features of cases (patients with HCC) and controls (patients with chronic liver disease, free of HCC). Continuous variables are presented as medians [interquartile range], categorical variables as frequencies (%).

	HCC (yes) *N* = 110	HCC (no) *N* = 330	*p* value
Age, years	74 [69-78]	74 [68-77]	0.208
Male gender, *N* (%)	67 (61)	201 (61)	0.788
Body mass index (kg/m^2^)	25 [23-28]	25 [22-29]	0.673
Diabetes, *N* (%)	28 (25)	53 (16)	0.033
History of alcohol abuse, *N* (%)	25 (23)	39 (12)	0.008
HBsAg, positive (%)	3 (3)	4 (1)	0.374
AST (U/L)	63 [43-93]	51 [34-87]	0.040
ALT (U/L)	54 [34-85]	56 [32-88]	0.633
Liver stiffness (kPa)	15.9 [12.0-21.8]	10.7 [6.8-16.6]	<0.001
AST to platelet ratio index	1.36 [0.78-2.11]	0.87 [0.45-1.61]	<0.001

Abbreviations: HCC: hepatocellular carcinoma; HCV: hepatitis C virus; HBsAg: hepatitis B virus surface antigen; AST: aspartate aminotransferase; ALT: alanine aminotransferase.

**Table 2:** Genotype and allele frequencies of the genes of interest. The *P* values test the hypothesis of deviation from Hardy-Weinberg equilibrium.

PNPLA3 (*N* = 440)		HSD17B13 (*N* = 440)	
C/C	239 (0.54)	T/T	243 (0.55)
C/G	171 (0.39)	T/TA	176 (0.40)
G/G	30 (0.07)	TA/TA	21 (0.05)
G/∗	201 (0.46)	TA/∗	197 (0.45)
C	649 (0.74)	T	662 (0.75)
G	231 (0.26)	TA	218 (0.25)
HWE	0.94	HWE	0.13

Abbreviations: PNPLA3: patatin-like phospholipase domain containing 3; HSD17B13: 17*β*-hydroxysteroid dehydrogenase type 13; HWE: Hardy-Weinberg equilibrium.

**Table 3:** Conditional logistic regression model of predictors of hepatocellular carcinoma. High-risk genetic combination defined as being at the same time the carrier of the variant rs738409 G allele and of the major allele rs72613567 allele.

Covariate	*N*	Odds ratio	95% confidence interval	*p* value
High-risk genetic combination				
No	331	(Ref.)	(Ref.)	(Ref.)
Yes	109	1.96	1.19-3.21	0.008
History of alcohol abuse				
No	376	(Ref.)	(Ref.)	(Ref.)
Yes	64	2.47	1.32-4.64	0.005
Diabetes mellitus				
No	359	(Ref.)	(Ref.)	(Ref.)
Yes	81	1.66	0.96-2.88	0.067
APRI > 1				
No	226	(Ref.)	(Ref.)	(Ref.)
Yes	214	2.41	1.53-3.81	<0.001

Abbreviations: APRI: AST to platelet ratio index; Ref. = reference.

## Data Availability

The data used to support the findings of this study are available from the corresponding author upon request.

## Abbreviations

HCC: Hepatocellular carcinoma
SNP: Single-nucleotide polymorphism
HCV: Hepatitis C virus
BCLC: Barcelona Clinic Liver Cancer
EASL: European Association for the Study of the Liver
LS: Liver stiffness
AST: Aspartate aminotransferase
APRI: AST to platelet ratio index
PCR: Polymerase chain reaction
CLD: Chronic liver diseases
NAFLD: Nonalcoholic fatty liver disease.

## Supplementary Materials

Supplementary Materials Supplementary Table 1: comparison of main demographic, anthropometric, and clinical features in patients without HCC, based on METAVIR stage. Continuous variables are presented as medians [interquartile range], and categorical variables as frequencies (%). Abbreviations—HCV: hepatitis C virus; AST: aspartate aminotransferase; ALT: alanine aminotransferase.

## References

[1] Pimpin L., Cortez-Pinto H., Negro F. (2018). Burden of liver disease in Europe: epidemiology and analysis of risk factors to identify prevention policies. *Journal of Hepatology*.

[2] Global Burden of Disease Liver Cancer Collaboration (2017). The burden of primary liver cancer and underlying etiologies from 1990 to 2015 at the global, regional, and national level: results from the Global Burden of Disease Study 2015. *JAMA Oncology*.

[3] Trépo E., Romeo S., Zucman-Rossi J., Nahon P. (2016). PNPLA3 gene in liver diseases. *Journal of Hepatology*.

[4] Ali M., Yopp A., Gopal P. (2016). A variant in *PNPLA3* associated with fibrosis progression but not hepatocellular carcinoma in patients with hepatitis C virus infection. *Clinical Gastroenterology and Hepatology*.

[5] Li J. F., Zheng E. Q., Xie M. (2019). Association between rs738409 polymorphism in patatin-like phospholipase domain-containing protein 3 (PNPLA3) gene and hepatocellular carcinoma susceptibility: evidence from case-control studies. *Gene*.

[6] Abul-Husn N. S., Cheng X., Li A. H. (2018). A protein-truncating *HSD17B13* variant and protection from chronic liver disease. *The New England Journal of Medicine*.

[7] Bellan M., Colletta C., Barbaglia M. N. (2019). Severity of nonalcoholic fatty liver disease in type 2 diabetes mellitus: relationship between nongenetic factors and PNPLA3/HSD17B13 polymorphisms. *Diabetes and Metabolism Journal*.

[8] Ma Y., Belyaeva O. V., Brown P. M. (2019). 17-Beta hydroxysteroid dehydrogenase 13 is a hepatic retinol dehydrogenase associated with histological features of nonalcoholic fatty liver disease. *Hepatology*.

[9] Marchais-Oberwinkler S., Henn C., Möller G. (2011). 17*β*-Hydroxysteroid dehydrogenases (17*β*-HSDs) as therapeutic targets: protein structures, functions, and recent progress in inhibitor development. *The Journal of Steroid Biochemistry and Molecular Biology*.

[10] Yang J., Trépo E., Nahon P. (2019). A 17-Beta-hydroxysteroid d.ehydrogenase 13 variant protects from hepatocellular carcinoma development in alcoholic liver disease. *Hepatology*.

[11] Rafiq N., Younossi Z. M. (2008). Interaction of metabolic syndrome, nonalcoholic fatty liver disease and chronic hepatitis C. *Expert Review of Gastroenterology & Hepatology*.

[12] Galle P. R., Forner A., Llovet J. M. (2018). EASL clinical practice guidelines: management of hepatocellular carcinoma. *Journal of Hepatology*.

[13] Forner A., Vilana R., Ayuso C. (2008). Diagnosis of hepatic nodules 20 mm or smaller in cirrhosis: prospective validation of the noninvasive diagnostic criteria for hepatocellular carcinoma. *Hepatology*.

[14] Afdhal N. H., Bacon B. R., Patel K. (2015). Accuracy of Fibroscan, compared with histology, in analysis of liver fibrosis in patients with hepatitis B or C: a United States Multicenter Study. *Clinical Gastroenterology and Hepatology*.

[15] Castera L., Forns X., Alberti A. (2008). Non-invasive evaluation of liver fibrosis using transient elastography. *Journal of Hepatology*.

[16] Lin Z. H., Xin Y. N., Dong Q. J. (2011). Performance of the aspartate aminotransferase-to-platelet ratio index for the staging of hepatitis C-related fibrosis: an updated meta-analysis. *Hepatology*.

[17] International Alliance for Responsible Drinking (IARD) Drinking guidelines: general population. http://www.iard.org/resources/drinking-guidelines-general-population/.

[18] Crispo A., Barba M., Malvezzi M., Ciliberto G., Montella M. (2014). Mortality trend for liver cancer in a hyperendemic area of hepatitis C virus infection in southern Italy: join-point analysis and comparison with European and Italian data. *European Journal of Gastroenterology & Hepatology*.

[19] Li D. K., Chung R. T., Law M. (2019). Overview of direct-acting antiviral drugs and drug resistance of hepatitis C virus. *Hepatitis C Virus Protocols*.

[20] Pawlotsky J. M., Negro F., Aghemo A. (2018). EASL recommendations on treatment of hepatitis C 2018. *Journal of Hepatology*.

[21] El-Serag H. B. (2012). Epidemiology of viral hepatitis and hepatocellular carcinoma. *Gastroenterology*.

[22] Nahon P., Zucman-Rossi J. (2012). Single nucleotide polymorphisms and risk of hepatocellular carcinoma in cirrhosis. *Journal of Hepatology*.

[23] Fan J. H., Xiang M. Q., Li Q. L., Shi H. T., Guo J. J. (2016). PNPLA3 rs738409 polymorphism associated with hepatic steatosis and advanced fibrosis in patients with chronic hepatitis C virus: a meta-analysis. *Gut Liver*.

[24] Mitsche M. A., Hobbs H. H., Cohen J. C. (2018). Patatin-like phospholipase domain–containing protein 3 promotes transfer of essential fatty acids from triglycerides to phospholipids in hepatic lipid droplets. *The Journal of Biological Chemistry*.

[25] BasuRay S., Smagris E., Cohen J. C., Hobbs H. H. (2017). The PNPLA3 variant associated with fatty liver disease (I148M) accumulates on lipid droplets by evading ubiquitylation. *Hepatology*.

[26] Pirazzi C., Adiels M., Burza M. A. (2012). Patatin-like phospholipase domain-containing 3 (PNPLA3) I148M (rs738409) affects hepatic VLDL secretion in humans and in vitro. *Journal of Hepatology*.

[27] Pirazzi C., Valenti L., Motta B. M. (2014). PNPLA3 has retinyl-palmitate lipase activity in human hepatic stellate cells. *Human Molecular Genetics*.

[28] Guyot E., Sutton A., Rufat P. (2013). PNPLA3 rs738409, hepatocellular carcinoma occurrence and risk model prediction in patients with cirrhosis. *Journal of Hepatology*.

[29] Grimaudo S., Pipitone R. M., Pennisi G. (2020). Association between *PNPLA3* rs*738409* C>G variant and liver-related outcomes in patients with nonalcoholic fatty liver disease. *Clinical Gastroenterology and Hepatology*.

[30] Seko Y., Sumida Y., Tanaka S. (2017). Development of hepatocellular carcinoma in Japanese patients with biopsy-proven non-alcoholic fatty liver disease: association between PNPLA3 genotype and hepatocarcinogenesis/fibrosis progression. *Hepatology Research*.

[31] Liu Y. L., Patman G. L., Leathart J. B. (2014). Carriage of the *PNPLA3* rs738409 C > G polymorphism confers an increased risk of non-alcoholic fatty liver disease associated hepatocellular carcinoma. *Journal of Hepatology*.

[32] Khlaiphuengsin A., Kiatbumrung R., Payungporn S., Pinjaroen N., Tangkijvanich P. (2015). Association of PNPLA3 polymorphism with hepatocellular carcinoma development and prognosis in viral and non-viral chronic liver diseases. *Asian Pacific Journal of Cancer Prevention*.

[33] Walker A. J., Peacock C. J., Pedergnana V., STOP‐HCV Consortium, Irving W. L. (2018). Host genetic factors associated with hepatocellular carcinoma in patients with hepatitis C virus infection: a systematic review. *Journal of Viral Hepatitis*.

[34] Ezzikouri S., Alaoui R., Tazi S. (2014). The adiponutrin I148M variant is a risk factor for HCV-associated liver cancer in North-African patients. *Infection, Genetics and Evolution*.

[35] Corradini S. G., Burza M. A., Molinaro A., Romeo S. (2011). Patatin-like phospholipase domain containing 3 sequence variant and hepatocellular carcinoma. *Hepatology*.

[36] Trépo E., Nahon P., Bontempi G. (2014). Association between the *PNPLA3* (rs738409 C > G) variant and hepatocellular carcinoma: evidence from a meta-analysis of individual participant data. *Hepatology*.

[37] Balasus D., Way M., Fusilli C. (2016). The association of variants in *PNPLA3* and *GRP78* and the risk of developing hepatocellular carcinoma in an Italian population. *Oncotarget*.

[38] Sato M., Kato N., Tateishi R. (2014). Impact of *PNPLA3* polymorphisms on the development of hepatocellular carcinoma in patients with chronic hepatitis C virus infection. *Hepatology Research*.

[39] Takeuchi Y., Ikeda F., Moritou Y. (2013). The impact of patatin-like phospholipase domain-containing protein 3 polymorphism on hepatocellular carcinoma prognosis. *Journal of Gastroenterology*.

[40] Yang J., Trépo E., Nahon P. (2018). PNPLA3 and TM6SF2 variants as risk factors of hepatocellular carcinoma across various etiologies and severity of underlying liver diseases. *International Journal of Cancer*.

[41] Yen Y. H., Tsai M. C., Wu C. K. (2018). Association between PNPLA3 (rs738409 C>G) variant and hepatocellular carcinoma in Asian chronic hepatitis C patients: a longitudinal study. *Journal of the Formosan Medical Association*.

[42] Hai H., Tamori A., Thuy L. T. T. (2017). Polymorphisms in *MICA*, but not in *DEPDC5*, *HCP5* or *PNPLA3*, are associated with chronic hepatitis C-related hepatocellular carcinoma. *Scientific Reports*.

[43] Huang Z., Guo X., Zhang G., Liang L., Nong B. (2019). Correlation between *PNPLA3* rs738409 polymorphism and hepatocellular carcinoma: a meta-analysis of 10,330 subjects. *The International Journal of Biological Markers*.

[44] About F., Abel L., Cobat A. (2018). HCV-associated liver fibrosis and HSD17B13. *The New England Journal of Medicine*.

[45] Su W., Wang Y., Jia X. (2014). Comparative proteomic study reveals 17*β*-HSD13 as a pathogenic protein in nonalcoholic fatty liver disease. *Proceedings of the National Academy of Sciences of the United States of America*.

[46] Pirola C. J., Garaycoechea M., Flichman D. (2019). Splice variant rs72613567 prevents worst histologic outcomes in patients with nonalcoholic fatty liver disease. *Journal of Lipid Research*.

[47] Szabo G., Saha B., Bukong T. N. (2015). Alcohol and HCV: implications for liver cancer. *Advances in Experimental Medicine and Biology*.

[48] Iida-Ueno A., Enomoto M., Tamori A., Kawada N. (2017). Hepatitis B virus infection and alcohol consumption. *World Journal of Gastroenterology*.

