# Supplementary material for: Interplay of PNPLA3 and HSD17B13 Variants in Modulating the Risk of Hepatocellular Carcinoma among Hepatitis C Patients
Source: Gastroenterol Res Pract. 2020 Apr 24;2020:4216451. doi: 10.1155/2020/4216451 (PMC7196159; doi:10.1155/2020/4216451)
Supplement: Supplementary Materials — Supplementary Table 1: comparison of main demographic, anthropometric, and clinical features in patients without HCC, based on METAVIR stage. Continuous variables are presented as medians [interquartile range], and categorical variables as frequencies (%). Abbreviations—HCV: hepatitis C virus; AST: aspartate aminotransferase; ALT: alanine aminotransferase. [file 4216451.f1.docx]

**Supplementary Table 1. Comparison of main demographic, anthropometric and clinical features in patients without HCC, based on METAVIR stage.** Continuous variables are presented as medians [interquartile range], categorical variables as frequencies (%). Abbreviations: HCV; hepatitis C virus; AST, aspartate aminotransferase; ALT, alanine aminotransferase.

|  | F0-F1  N.=92 | F2-F3  N.=110 | F4  N.=128 | p |
| --- | --- | --- | --- | --- |
| Age, years | 73 [68-77] | 74 [67-79] | 74 [68-77] | 0.561 |
| Male gender, N. (%) | 53 (58) | 75 (68) | 73 (57) | 1.000 |
| Body mass index, kg/m^2^ | 25 [22-28] | 25 [23-28] | 26 [22-29] | 0.243 |
| Diabetes, N. (%) | 10 (11) | 19 (17) | 24 (19) | 0.128 |
| Alcohol abuse, N. (%) | 5 (5) | 16 (15) | 18 (14) | 0.073 |
| HCV genotype 1, N. (%) | 43 (47) | 62 (57) | 81 (63) | 0.015 |
| AST, U/L | 32 [26-45] | 53 [36-86] | 72 [50-104] | <0.001 |
| ALT, U/L | 32 [21-54] | 62 [38-99] | 73 [45-118] | <0.001 |
| Liver stiffness, kPa | 5.8 [4.7-6.4] | 9.9 [8.2-11.3] | 20 [15.2-27] | <0.001 |
| AST to platelets ratio index | 0.43 [0.32-0.58] | 0.79 [0.46-1.28] | 1.59 [1.00-2.55] | <0.001 |
